# Forensics System for Internet of Vehicles Based on Post-Quantum Blockchain

**DOI:** 10.3390/s25196038

**Published:** 2025-10-01

**Authors:** Zheng Zhang, Zehao Cao, Yongshun Wang

**Affiliations:** 1 School of Electronic and Information Engineering, Lanzhou Jiaotong University, Lanzhou 730070, China; 2 School of Architecture and Urban Planning, Lanzhou Jiaotong University, Lanzhou 730070, China; 3 School of Architecture, Tianjin University, Tianjin 300072, China

**Keywords:** Internet of Vehicles, blockchain, privacy protection, forensics, lattice cryptography, ring signature

## Abstract

Internet of Vehicles (IoV) serves as the data support for intelligent transportation systems, and the information security of the IoV is of paramount importance. In view of the problems of centralized processing, easy information leakage, and weak anti-interference ability in traditional vehicle networking systems, this paper proposes a blockchain architecture suitable for IoV forensics scenario. By leveraging the decentralized, distributed storage and tamper-proof capabilities of blockchain, it solves the privacy protection and data security issues of the system. Considering the threat of quantum computing to the encryption technology in traditional blockchain, this paper integrates lattice cryptography and ring signatures into digital signature technology, achieving privacy protection and traceability of the signer’s identity. To enhance the efficiency of lattice-based cryptographic algorithms, the DualRing technology is introduced, which reduces the computational time and storage consumption of ring signatures. Theoretical analysis has proved the correctness, anonymity, unlinkability, and traceability of the proposed scheme, which is applicable to the IoV forensics system. Simulation comparisons demonstrated that the proposed scheme significantly improves computational efficiency and reduces storage overhead. When the number of ring members is 256, the signature and verification times require only 65.76 ms and 21.46 ms, respectively.

## 1. Introduction

With the continuous upgrading of intelligent vehicle hardware and software, the widespread adoption of 6G networks, and the development of intelligent transportation systems, Internet of Vehicles (IoV) technology has gradually become a research hotspot in the intelligent era. Accident investigation and evidence preservation are one of its key applications. The vehicle’s on-board unit records key details like speed, brake status, throttle status, and driving path. This data can be essential for determining the cause of an accident. Also, useful information from nearby vehicles’ dashcams, sensors, and infrastructure at the accident scene can be key evidence.

Vehicular Ad Hoc Networks (VANETs) are wireless self-organizing communication networks designed for vehicles. Vehicles can use wireless technology to exchange information with other vehicles (V2V, Vehicle-to-Vehicle) or roadside devices (V2I, Vehicle-to-Infrastructure). VANETs upload the collected evidence to the cloud, making it accessible for real-time queries by traffic management, insurance, and court departments. Traditional vehicle networking systems use cloud storage, which poses risks of single points of failure and data breaches [1]. They also struggle to meet the needs of high-dynamic, low-latency, and distributed environments. Blockchain, as a decentralized, anonymous, transparent, traceable, and tamper-proof distributed ledger technology, uses cryptography to ensure data security. This can solve issues like communication security, single points of failure, and data storage in the IoV. Other databases, like LedgerDB [2] and VeDB [3], provide tamper-resistant features and high performance. However, they use a centralized structure and depend on the operator to manage the data.

Research on vehicle accident evidence collection based on blockchain has become a hot topic. In terms of system design, Davydov et al. [4] proposed an online and offline accident detection model based on blockchain, Pujol et al. [5] proposed an accident prevention framework for IoV based on blockchain, and Yao et al. [6] proposed a model to determine vehicle accident liability for IoV using lightweight blockchain. In terms of technology integration, Philip et al. [7] utilized smart contract technology to collect and manage evidence, proposing a conceptual evidence management framework. In studies [8,9], Guo et al. used dynamic alliance consensus in self-driving systems to gather and record accident evidence.

In terms of data security, Vangala et al. [10] developed a new blockchain system for authenticating certificates. This helps detect and notify about vehicle accidents in smart transportation systems. Dwivedi et al. [11] proposed a protocol that uses blockchain mechanisms to protect event information and vehicle authentication. Xie et al. [12] proposed a third-party traffic accident handling protocol and designed a dynamic pseudo-identity strategy to protect vehicle privacy. The multi-level blockchain framework designed by Lin [13] adopts the elliptic curve digital signature algorithm to achieve identity verification and non-repudiation execution.

The above-mentioned solutions achieve accident detection, recording, and processing in the IoV through improvements to blockchain technology. However, they overlook the risk that quantum computing poses to blockchain encryption and data security. Although quantum computers are still in the research stage, it has been confirmed that their computing power can crack traditional encryption algorithms [14]. Shakib et al. [15] proved that identity impersonation attacks are feasible in blockchain-based IoV systems, which threaten the security of data. They also stressed the need for quantum-secure blockchains in IoV. Like blockchain, the SecuDB [16] database protects privacy by using a trusted execution environment. However, its security relies heavily on the trustworthiness of the hardware manufacturer.

At present, lattice-based post-quantum blockchain technology is the core solution for ensuring data security. This technology has already found some applications in the field of IoV. Gupta et al. [17] created a new certificateless data authentication protocol. It uses lattice cryptography to defend against quantum attacks. This improves the security of wireless communication networks in the IoV. Zhang et al. [18] suggested a grid signature scheme for blockchain authentication. This method ensures secure energy transactions and safe information sharing in the IoV. However, in the field of electronic forensics for IoV, research on post-quantum blockchain is relatively scarce. This paper addresses the application gap in forensics by integrating blockchain technology into the IoV forensics architecture. It improves the lattice cryptographic algorithm and designs a system that can resist quantum threats, protect vehicle privacy, and prevent information tampering.

Our main contributions are detailed as follows:

(1) Research the blockchain architecture applicable to the field of electronic forensics in the IoV. Use road side units and cloud servers as consensus nodes in a consortium chain to achieve distributed storage and computing, reducing the computing pressure on the system while ensuring data security.

(2) By combining ring signature technology with lattice cryptography, we designed a new signature algorithm. This algorithm meets several key requirements: anonymous evidence upload, evidence integrity, signature unlinkability, identity traceability, and resistance to quantum attacks.

(3) To address the issues of large storage space and high computational complexity in lattice cryptography. The algorithm in this paper has been significantly improved in terms of memory space and computational efficiency. With a ring size of 256, the signature time is only 65.76 ms. This feature makes it suitable for small and medium ring signature schemes, ranging from 5 to 1000. Its application possibilities are extensive.

The structure of this article is as follows: In Section 2, we introduce the blockchain-based IoV system. This section provides an overview of blockchain technology, describes the theoretical basis of cryptography, and introduces the definition and security model of the ring signature algorithm. In Section 3, we present the detailed process of the signature algorithm and elucidate its role within the blockchain system. In Section 4, we verify the correctness and security of the proposed scheme. In Section 5, we describe the parameter settings and present simulation comparisons for the algorithm. Finally, Section 6 summarizes the achievements of the article.

## 2. Preliminary Knowledge

In this section, we provide a detailed introduction to the blockchain-based IoV system, lattice cryptography algorithms, and traceable ring signature.

### 2.1. Blockchain-Based IoV System

The rapid rise of intelligent transportation technology and new energy vehicles has provided a huge development prospect for IoV technology. As an important application branch of the Internet of Things, the IoV is a heterogeneous network composed of VANETs and mobile communication networks. While the vehicle operates, it faces issues like fast node movement, unstable networks, scattered nodes, data delays, and low storage capacity. More importantly, there is a risk of information security leakage. Research on blockchain for IoV security includes key management, authentication methods, access control, trust management, and privacy protection [19]. In general, the composition of VANETs can be divided into three main parts:

TA (Trusted Authority) is responsible for vehicle registration and identity information storage. It is known as the authoritative department in VANETs and can act as an administrator and a trusted third party. The main functions of TA are to generate system parameters, vehicle keys, and track identities.

RSUs (Road Side Units) are fixed structures at crossroads. They have computing and storage power. Also, they act as signature verification nodes and consensus nodes in the blockchain system.

OBUs (On-Board Units) are storage, computing, and communication devices fixed on the vehicle. In the blockchain, the vehicle signs the information that needs to be uploaded and sends it to the nearby RSUs node.

In the blockchain-based IoV system, vehicles can generate signatures for their messages. They use encryption algorithms to encapsulate the messages, signatures, and other details into a transaction. After that, they send this transaction to nearby RSU nodes to verify the signature’s correctness. When an RSU node collects sufficient correct information, it can generate a block. This block consists of two parts: the block header and the block body.

**Figure 1:**
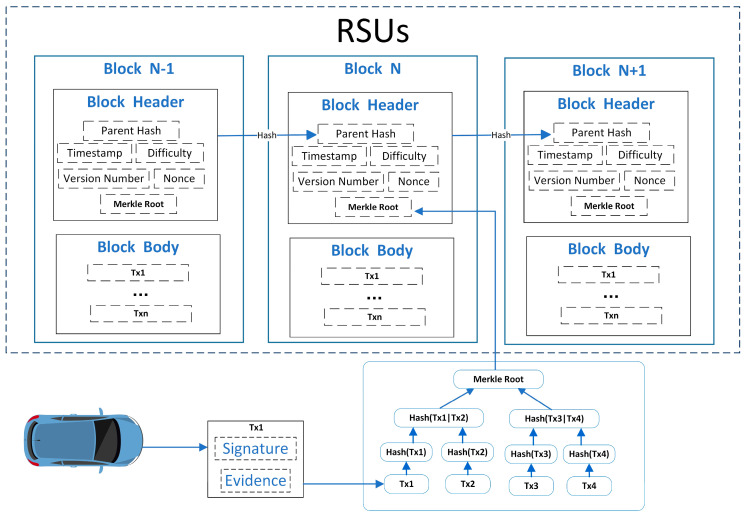
Blockchain Structure.

The block header is made up of three parts: (1) Parent Hash: This hash connects to the previous block. It used to form a chain structure and prevent the block data from being tampered with. (2) Basic Information: This section includes items like version, timestamp, difficulty target, and nonce. (3) Merkle Root: All transactions (Tx) are grouped and subjected to hash operations until a root hash value is formed, which is used to prevent the transaction information from being tampered with. Transactions are stored in the block body. The specific process is shown in Figure 1:

The RSUs node generates a block proposal and broadcasts it to other nodes for verification. Different consensus mechanisms have different operation speeds and applicable scenarios. The existing blockchains are mainly divided into three categories: public chains, consortium chains, and private chains. Public chains have the highest level of transparency and can achieve complete decentralization. However, their consensus mechanisms operate at a slower pace and require a large amount of computing power. This makes them unsuitable for latency-sensitive applications.

A consortium chain is a semi-open blockchain where only verified legitimate users can access and upload transactions. Different nodes have different levels of rights. Transaction throughput is significantly higher than that of public blockchains. It can reach up to 1000 transactions per second by using different consensus mechanisms. It is suitable for joint transaction settlements among different entities, which is more applicable to scenarios involving multi-party collaboration in the IoV [20]. A private chain is suitable for internal use within an institution or for individuals to set their own rules for accounting. The credibility of user nodes is relatively high, and transaction confirmation is faster and more efficient, but the degree of decentralization is not strong.

### 2.2. Lattice Cryptography

During the research process of quantum algorithms, scholars have discovered that quantum algorithms do not have any obvious advantages in difficult problems on lattices. Cryptographic techniques based on lattices are known as post-quantum cryptographic techniques. The following is a brief introduction to the relevant concepts.

#### 2.2.1. Notation

For an odd prime q, let ${\mathbb{Z}}_{q}$ represent the integers modulo q, and denote as elements in the interval $\left[-\left(q-1\right)/2,\left(q-1\right)/2\right]$. We use $R=\mathbb{Z}\left[x\right]/\left(x^{d}+1\right)$ and $R_{q}={\mathbb{Z}}_{q}\left[x\right]/\left(x^{d}+1\right)$ to denote the polynomial rings, where d is a power of 2 [21].

Let $f\left(x\right)=f_{0}+f_{1}\cdot x+\ldots +f_{d-1}\cdot x^{d-1}\in R$, and its *l*-norm be defined as

(1) $$l_{1}:{\left\|f\right\|}_{1}=\sum_{i=0}^{d-1}\left|f_{i}\right|,\;l_{2}:{\left\|f\right\|}_{2}={\left({\sum_{i=0}^{d-1}\left|f_{i}\right|}^{2}\right)}^{1/2},\;l_{\infty }:{\left\|f\right\|}_{\infty }=\underset{0\leq i\leq d-1}{\max}\left|f_{i}\right|$$

Define the set as $S_{\beta }=\left\{f\in R_{q},\;{\left\|f\right\|}_{\infty }\leq \beta \right\}$, and the challenge space as $D=\left\{g\in R_{q},\;{\left\|g\right\|}_{\infty }\leq 1\right\}$ with more than $3^{d}$ elements, which is a commutative group under addition mod 3.

#### 2.2.2. Lattice and Hard Problems

**Definition** **1.** (Lattice)**.** *Let $B=\left\{b_{1},\ldots \;,b_{m}\right\}\in {\mathbb{R}}^{n\times m}$ be a set of linearly independent m-dimensional vectors. An m-dimensional lattice Λ is defined as*

(2) $$\Lambda =L\left(B\right)=\left\{\sum_{i=1}^{m}a_{i}b_{i}:a_{i}\in \mathbb{Z}\right\}$$

*where B is a basis of $\Lambda =L\left(B\right)$, m and n are the dimensions and rank of Λ.*

Next, we will introduce the Module Short Integer Solution (MSIS) and Module Learning with Errors (MLWE) problems [22]. These problems cannot be solved in polynomial time with non-negligible probability. The algorithm proposed in this paper is based on the difficulty of the following two types of problems to ensure system security.

**Definition** **2.** ($MSIS_{m,n,q,\beta }$ Problem)**.** *Given a matrix $A\leftarrow R_{q}^{n\times m}$, find a nonzero vector $x\in R^{m}$ such that Ax=0modq and ${\left\|x\right\|}_{2}\leq \beta$. The MISIS (inhomogeneous definition) is given a matrix $A\leftarrow R_{q}^{n\times m}$ and a vector $b\in R_{q}^{n}$, asks to find a nonzero vector $x\in R^{m}$ such that Ax=bmodq and ${\left\|x\right\|}_{2}\leq \beta$. “Hermite Normal Form” is an important definition in the MSIS problem; it can change $A\leftarrow R_{q}^{n\times m}$ to $A=\left[A^{\prime }∥I_{n}\right]$ and $A^{\prime }\leftarrow R_{q}^{n\times \left(m-n\right)}$. This variant is as hard as the MSIS problem given above [23].*

**Definition** **3.** ($MLWE_{m,n,q,\beta }$ Problem)**.** *Given a matrix $A\leftarrow R_{q}^{n\times m}$, let $b=As+e\in R_{q}^{n}$, where $s\leftarrow S_{\beta }^{m}$ and error vector $e\leftarrow S_{\beta }^{n}$. The Decisional MLWE (D-MLWE) asks to distinguish the distribution of $\left(A,b\right)$ and $\left(A,u\right)$, where $u\in R_{q}^{n}$. The Search MLWE (S-MLWE) asks to find a non-zero $s^{\prime }\leftarrow S_{\beta }^{m}$ such that $b=As^{\prime }+e$ and ${\left\|s^{\prime }\right\|}_{\infty }\leq \beta$ [24]. According to references [23,25], in the MLWE problem, β=1 can be taken, which is more suitable for lattice-based cryptosystems.*

#### 2.2.3. MLWE-Based Public Key Cryptosystem

In 2009, Regev proposed the first LWE-based PKC (Public Key Cryptosystem) [26]. In 2013, Lyubashevsky improved upon it and presented the RLWE (Ring Learning with Errors)-based cryptosystem [27]. The MLWE-based cryptosystem proposed in this paper can be regarded as an improvement from RLWE to MLWE, or the non-compressed IND-CPA-secure encryption algorithm of Kyber as described in [28].

$PP\leftarrow Setup\left(1^{\lambda }\right)$: Let n, v, d, q be positive integer parameters, n and v are the row and column vectors of the matrix. $M={\left\{0,1\right\}}^{d}$ is the message space. Each message μ∈M can be regarded as the 0–1 coefficient of the polynomial R. $\chi \in R_{q}$ is a distribution with elements that have a small infinity norm. λ is the security parameter. 「 」 is a rounding-off symbol, and 「x」 denotes rounding x to the closest integer, with ties being rounded up. $A^{T}$ denotes the transpose of matrix A.

$\left(pk,sk\right)\leftarrow KeyGen\left(PP\right)$: Randomly select two small elements $\left(s,e\right)\leftarrow \chi^{v}\times \chi^{n}$ and a random matrix $A\leftarrow R_{q}^{n\times v}$, and compute b=As+e. The public and private keys can be obtained, respectively, as $pk=\left(A,b\right)$, $sk=\left(s,e\right)$.

$C\leftarrow Enc\left(\mu ,pk\right)$: The *d*-bit message μ needs to be encrypted, chooses three random small elements $\left(r,\varepsilon_{1},\varepsilon_{2}\right)\leftarrow \chi^{n}\times \chi^{v}\times \chi$, and computes the ciphertext:

(3) $$C=\left(C_{1},C_{2}\right)=\left(A^{T}r+\varepsilon_{1},b^{T}r+\varepsilon_{2}+「q/2」\mu \right)$$

$\mu \leftarrow Dec\left(C,s\right)$: After receiving the ciphertext C, the message μ can be decrypted by using the private key. The specific algorithm is

(4) $$\begin{array}{cc} \mu^{\prime } & =C^{2}-s^{T}C_{1}\\ & =b^{T}r+\varepsilon_{2}+「q/2」\mu -s^{T}A^{T}r-s^{T}\varepsilon_{1}\\ & =\left(e^{T}r+\varepsilon_{2}-s^{T}\varepsilon_{1}\right)+「q/2」\mu \mathrm{mod}q \end{array}$$

$\mu^{\prime }$ denotes the calculation result. For an appropriate choice of parameters, the infinity of ${\left\|e^{T}r+\varepsilon_{2}-s^{T}\varepsilon_{1}\right\|}_{\infty }\leq 「q/4」$, so the bits of μ can be recovered by rounding each coefficient of $\mu^{\prime }$ back to either 0 or 「q/2」, whichever is closest modulo q [21].

When $S_{\beta }$ with an appropriate small β, the distribution of $S_{\beta }$ is consistent with that of χ, which is a binomial distribution or a uniform distribution. Then, the $MLWE_{v=n-1,n,q,\chi =S_{\beta }}$ based cryptosystem is pseudorandom. It meets IND-CPA (Indistinguishability under Chosen Plaintext Attack) security requirements [21].

### 2.3. Traceable Ring Signatures

A ring signature is a signature algorithm that can achieve anonymity without the collaboration of ring members. Based on different functional attributes, it can be categorized into linkable ring signatures, threshold ring signatures, and traceable ring signatures. Since 2015, research on ring signatures based on traditional hard mathematical problems has gradually decreased. Meanwhile, improved schemes of lattice cryptography and multivariate cryptography, which can resist quantum attacks, have increased year by year [29]. Due to the high computational complexity of lattice-based cryptography, the above schemes all have problems such as a large signature size and a long operation time. This paper introduces a novel double-ring signature construction. It aims to boost computational efficiency and lower signature size.

#### 2.3.1. DualRing Signature Construction

**Figure 2:**
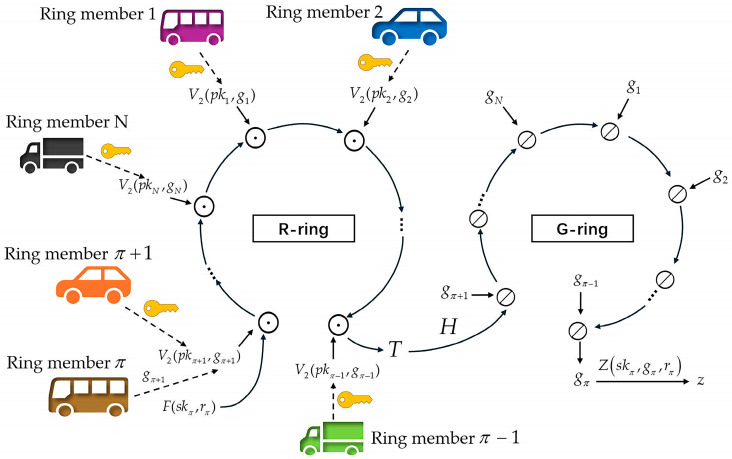
DualRing Signature Construction.

The ring signature scheme proposed in this paper is based on the new ring signature construction—DualRing proposed by Yuen et al. [25], which is built on the Type-T (Three-Move type) signature [30]. The signer collects the public keys of nearby vehicles to form a ring L. In Figure 2, vehicles in the IoV system are represented as ring members. The verifier can only confirm that the signature comes from one of the members in the set L, but does not know who the signer is. The more ring members there are, the stronger the concealment.

In [25], Yuen et al. constructed the DualRing signature using symbols ⊗ and ⊙ to represent two group exchange operations (⊗ represents modular addition, and ⊙ represents modular multiplication). ⊘ is the inverse of ⊗, and the verification function V(pk,z,g) is divided into two functions $V_{1}(z)$ and $V_{2}(pk,g)$, satisfying $V(pk,z,g)=V_{1}(z)⊙V_{2}(pk,g)$.

The signer π samples a random number $r_{\pi }$ and picks random challenges $g_{1},\ldots ,g_{\pi -1},g_{\pi +1},\ldots ,g_{N}$, and then uses a commit function F and $V_{2}$ to construct an R-ring to compute the commitment T.

(5) $$T=F(sk_{\pi },r_{\pi })⊙V_{2}(pk_{\pi +1},g_{\pi +1})⊙\ldots ⊙V_{2}(pk_{N},g_{N})⊙V_{2}(pk_{1},g_{1})\ldots ⊙V_{2}(pk_{\pi -1},g_{\pi -1})$$

Through a G-ring and a hash function H to compute the “missing” challenge $g_{\pi }=H\left(L,\mu ,T\right)⊘g_{\pi +1}⊘\ldots ⊘g_{N}⊘g_{1}\ldots ⊘g_{\pi -1}$. The final ring signature is $\sigma =\left(z,g_{1},\ldots ,g_{N}\right)$, with $z=Z\left(sk_{\pi },g_{\pi },r_{\pi }\right)$ generated by a response function Z. This signature is shorter than a general signature $\sigma =\left(z_{1},\ldots ,z_{N},g\right)$ because z is longer than g. So, this scheme can greatly reduce the signature size.

#### 2.3.2. Definition and Security Model of TRS

**Definition** **4.** *The Traceable Ring signature (TRS) scheme is defined by the following five polynomial-time algorithms:*

*$PP\leftarrow Setup\left(1^{\lambda }\right)$: The algorithm inputs a security parameter λ, and outputs public parameters PP, which are applicable to all users.*

*$\left(pk,sk\right)\leftarrow KeyGen\left(PP\right)$: The algorithm inputs public parameters PP and outputs a randomized public–private key pair $\left(pk,sk\right)$.*

*$\sigma \leftarrow Sign\left(PP,L,\mu ,sk_{\pi }\right)$: The algorithm inputs PP, the set of public keys $L=\left\{pk_{1},pk_{2}\ldots ,pk_{N}\right\}$, the message μ that needs to be signed, and the private key $sk_{\pi }$ of the signer π; it outputs a signature σ that contains a tracking tag C. We require the public key $pk_{\pi }\in L$.*

*$0/1\leftarrow Verify\left(PP,L,\mu ,\sigma \right)$: The algorithm inputs PP, the ring $L=\left\{pk_{1},pk_{2},\ldots ,pk_{N}\right\}$, and the signature pair $\left(\mu ,\sigma \right)$, then checks its validity. If it is valid, then it outputs 1; otherwise, it outputs 0.*

*$0/1\leftarrow Trace\left(PP,L,sk_{TA},\sigma \right)$: The algorithm inputs PP, the ring $L=\left\{pk_{1},pk_{2},\ldots ,pk_{N}\right\}$, the signature σ, and the TA’s secret key $sk_{TA}$. If the identity of the signer can be traced, then it outputs 1; otherwise, it outputs 0.*

The difference between the algorithm in this paper and the traceable ring signature algorithm lies in the fact that only TA can trace the identity of the signer. In electronic forensic systems, the signer’s identity is fully anonymous. It can’t be linked to other vehicles or organizations. The security definition of the traceable ring signature not only needs to meet the correctness, anonymity, and unforgeability of the ordinary ring signature, but also requires traceability by the TA.

**Definition** **5** (Correctness)**.** *This means that if an honest signer runs the signature algorithm properly, this signature has an overwhelming probability of being verified successfully.*

(6) $$\mathrm{Pr}\left[0\leftarrow Verify\left(PP,L,\mu ,\sigma \right):\begin{array}{c} PP\leftarrow Setup\left(1^{\lambda }\right)\\ \left(pk,sk\right)\leftarrow KeyGen\left(PP\right)\\ \sigma \leftarrow Sign\left(PP,L,\mu ,sk_{\pi }\right) \end{array}\right]\leq negl\left(\lambda \right)$$

Anonymity and unforgeability are the two main aspects of proving the security of ring signatures. During the proof process, a series of simulation games between the adversary A and the challenger S under the random oracle model is needed to prove anonymity and unforgeability. The adversary A can make the following two types of queries:

Key Generation Oracle $O_{K}$: The adversary A submit his $id_{i}$ to the challenger S. Then, S randomly generates a key pair $\left(pk_{i},sk_{i}\right)$ using the generation algorithm. S saves this information and returns it to A.

Signing Oracle $O_{S}$: On query, the adversary A inputs the ring $L=\left\{pk_{1},pk_{2},\ldots ,pk_{N}\right\}$, the signer’s public key $pk_{\pi }\in L$, and the message μ. The challenger S generates a signature σ according to the algorithm and returns it to A.

**Definition** **6** (Anonymity)**.** *It means that for a valid signature, it is impossible to guess the identity of the actual signer.*

Let $Adv_{A}^{anon}$ represent the probability of the adversary A winning. Consider the following game:

(1) Initialization: S runs the algorithm $Setup\left(1^{\lambda }\right)$ and inputs the security parameter λ to obtain the public parameters PP and the system private key $sk_{TA}$, and then sends PP to A.
(2) Query Phase: A could query $O_{K}$ and $O_{S}$ enough times. Through $O_{K}$ queries, A can obtain $L=\left\{pk_{1},pk_{2},\ldots ,pk_{N}\right\}$ and its corresponding private key.
(3) Forgery Phase: A inputs the ring $L=\left\{pk_{1},pk_{2},\ldots ,pk_{N}\right\}$ and the message μ, as well as two valid public keys, $pk_{i0}$ and $pk_{i1}$. S randomly selects $B\in \left\{0,1\right\}$, generates a signature σ according to the signature algorithm, and then returns it to A.
(4) Guessing Phase: A outputs B. If $B=B^{\prime }$, A wins the game.

According to the above definition of a game, the adversary’s winning advantage is

(7) $$Adv_{A}^{anon}=\left|\mathrm{Pr}\left[b=b^{\prime }\right]-\frac{1}{2}\right|$$

**Definition** **7** (Unforgeability)**.** *It means that a malicious user without the private key cannot generate verifiable signatures [21].*

The rules of the game are as follows:

(1) Initialization: S runs the algorithm $Setup\left(1^{\lambda }\right)$ and inputs the security parameter λ to obtain the public parameters PP and the system private key $sk_{TA}$, and then sends PP to A.
(2) Query Phase: A could query $O_{K}$ and $O_{S}$ enough times.
(3) Forgery Phase: A sends $\left(PP,L,\sigma ,\mu \right)$ to S. A wins if these conditions are met: S verifies the signature is valid; A hasn’t inquired about the private key of any member in ring L; A hasn’t asked about the signature of $\left(L,\mu \right)$.

According to the above definition of the game, the adversary’s winning advantage is

(8) $$Adv_{A}^{forge}=\mathrm{Pr}\left[A\;wins\;the\;game\right]$$

**Definition** **8** (TA traceability)**.** *An honest signer legally generates an identity-related tag by executing the signature algorithm, and this tag can only be traced back to the signer’s identity by the TA.*

## 3. Proposed Scheme

In this section, we will provide a detailed introduction to the blockchain system and its privacy encryption scheme applicable to IoV forensics. The blockchain framework is utilized to achieve tamper-proofing and distributed storage, while ring signature technology is used to enable anonymous vehicle forensics and tracking.

### 3.1. IoV Forensic System

The specific work of the system entities is introduced as follows:

(1) TA: Responsible for vehicle registration, generating public and private keys, and preserving the real identity. When the reference value of the signed message is relatively high, the relevant department requires TA to verify their identity. TA rewards or punishes the signatories of messages. If the TA verification is passed, it can be used as valid evidence, and certain rewards can be given to the vehicle according to the incentive mechanism. If the verification by the TA fails, the message will be invalid. Once false information is discovered, the TA will disclose the identity information and hold the person accountable.
(2) OBUs: At present, all intelligent vehicles are equipped with OBUs. When a vehicle receives a request for evidence collection, the OBU signs the information with a private key. Then, it sends this to the nearby RSU node. When issuing a task, RSUs will send the public key information of nearby vehicles. Help the vehicle form a temporary ring $L=\left\{pk_{1},pk_{2}\ldots ,pk_{N}\right\}$ and ensure the legitimacy of other ring members. Messages signed in this way can effectively prevent malicious nodes from tampering with information during communication. Evidence videos recorded by dashcams can be uploaded by OBUs. Basic equipment and sensors at the accident scene can record and calculate data. They can also upload this evidence automatically.
(3) RSUs: RSUs handle the evidence collection task. They share this task and the public key information of nearby vehicles, inviting them to join. They check the signatures from vehicles to confirm their validity and ensure all members are legitimate. After verifying, the information is saved. Once enough data is gathered, a new block is created and sent to each node for consensus verification, forming a distributed storage blockchain.
(4) Cloud servers: Each institution can set up a cloud server, which functions similarly to RSUs as consensus nodes and distributed storage nodes. However, the cloud server has enabled the query function to facilitate evidence collection by relevant institutions.
(5) Inquiry Institutions: Insurance companies, courts, vehicle management offices, and traffic police can issue evidence collection tasks via RSUs and use cloud servers for queries. Once valid evidence is found, the signature will be sent to the TA for identity verification. If the verification passes, it is considered that the message is available and 1 is returned; otherwise, 0 is returned to indicate that the message is not adopted. Throughout this process, except for the TA, no other entities know the identity of the signer. Inquiry institutions can store the valid information to form a new evidence chain.

**Figure 3:**
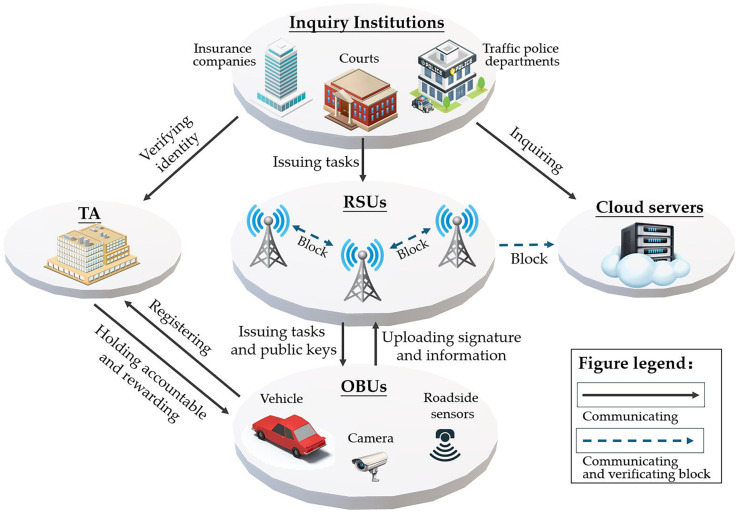
The Proposed IoV Forensics Framework.

As introduced in Section 2.1, this paper adopts a consortium chain structure, where all RSUs and the cloud servers form all the nodes for verification. The system design is shown in Figure 3:

### 3.2. Design Requirements

In the IoV forensics system, TA is a completely trusted department and uses wired transmission, which can ensure the security of information. However, RSUs and OBUs use wireless transmission technology, and the signed information will be released externally. To prevent malicious vehicles from stealing and tracking vehicle information or releasing false information, the forensics system must meet these requirements:

Identity privacy: To avoid retaliation, the information of vehicle users who provide evidence should be completely confidential. The vehicle that releases information should achieve complete anonymity except for TA.

Traceability: When malicious users share false information, it is also necessary for TA to authenticate and track user identities. This way, they can reward users who post valid information and hold accountable those who spread falsehoods.

Message integrity: Ensuring that messages are not tampered with during transmission. The blockchain’s transaction encryption keeps data safe from tampering. Its distributed storage can prevent message leakage and tampering caused by single-point failures.

Unlinkability: If the signature information is linkable, vehicle users may draw attention. When a vehicle frequently shares evidence, it may be seen as a highly credible node. Malicious nodes can associate vehicle activities with transmitted messages, which are not conducive to users’ evidence collection work.

Efficiency: While ensuring security, it is also necessary to consider issues such as computing speed and storage capacity. Try to reduce the computing pressure on OBUs and RSUs as much as possible.

### 3.3. Algorithm Design

$PP\leftarrow Setup\left(1^{\lambda }\right)$: TA initializes the VANETs system, sets the system parameters n and m to be positive integers, q is a prime number, and q≥3. Take v=m−n, random sample of a matrix $A^{\prime }\leftarrow R_{q}^{n\times v}$, according to the definition of “Hermite normal form”, it can be obtained that $A=\left[A^{\prime }∥I_{n}\right]$ and $A\leftarrow R_{q}^{n\times m}$. TA randomly selects $sk_{TA}=\left(s,e\right)\leftarrow S_{\beta }^{v}\times S_{\beta }^{n}$ as its private key, computes $b=A^{\prime }s+e=A\left[\begin{array}{c} s\\ e \end{array}\right]=A\cdot sk_{TA}$ to generate the public key $pk_{TA}=\left(A,b\right)$. After selecting two hash functions, $H:{\left\{0,1\right\}}^{*}\to D$ and $H^{\prime }:\left(\omega \right)\to {\left\{0,1\right\}}^{d/2}$, the system parameter $PP=\left\{n,m,q,A,b,H,H^{\prime }\right\}$ is finally obtained. The relevant parameters and definitions are shown in Table 1.

$\left(pk_{i},sk_{i}\right)\leftarrow KeyGen\left(PP\right)$: User i sends its own $id_{i}$ to TA to apply for the generation of public and private keys. TA randomly selects $sk_{i}=\left(s_{i},e_{i}\right)\leftarrow S_{\beta }^{v}\times S_{\beta }^{n}$ as the user’s private key and computes $pk_{i}=A^{\prime }s_{i}+e_{i}=A\cdot sk_{i}$ as the user’s public key. Generate the identity tag $id_{si}=\left[id_{i}|id_{s}\right]$ by computing $id_{s}=H^{\prime }\left(sk_{\pi }\right)\in {\left\{0,1\right\}}^{d/2}$. TA sends $\left(pk_{i},sk_{i},id_{si}\right)$ to the vehicle in a secure manner and saves the information in the registration information form. Once the vehicle violates regulations or engages in illegal activities, the real information of the vehicle can be queried through TA.

$\sigma \leftarrow Sign\left(PP,L,\mu ,sk_{\pi },id_{s\pi }\right)$: To prevent the identity information from being traced due to the public key, this paper adopts the ring signature method to conduct a secondary concealment of the identity. The signing vehicle receives the public key information of nearby vehicles, and then a temporary ring $L=\left\{pk_{1},pk_{2},\ldots ,pk_{N}\right\}$, $pk_{\pi }\in L$ is formed. The message that needs to be signed is $\mu \in {\left\{0,1\right\}}^{*}$. The signing process is as follows:

(1) The user π randomly selects three small elements $\left(r,\varepsilon_{1},\varepsilon_{2}\right)\leftarrow S_{\beta }^{n}\times S_{\beta }^{v}\times S_{\beta }$, and computes a tracking tag $C=\left(C_{1},C_{2}\right)=\left({A^{\prime }}^{T}r+\varepsilon_{1},b^{T}r+\varepsilon_{2}+q/2\cdot id_{s\pi }\right)$.
(2) For $i\in \left[N\right]\backslash \left\{\pi \right\}$, samples $y\leftarrow S_{md^{2}}^{m},g_{i}\leftarrow D$.
(3) Computes $t_{0}=Ay+\sum_{i\in \left[N\right]\backslash \left\{\pi \right\}}pk_{i}\cdot g_{i}$.
(4) Computes $g=H\left(L,C,\mu ,t_{0}\right)$ and $g_{\pi }=g⊘\left(g_{1}⊗\ldots g_{\pi -1}⊗g_{\pi +1}\ldots ⊗g_{N}\right)$.
(5) Computes $z=sk_{\pi }g_{\pi }-y$.

If $z\leftarrow S_{md^{2}-d}^{m}$, then output the signature $\sigma =\left(C,z,g_{1},\ldots g_{N}\right)$. Otherwise, return to (2) and start over.

$0/1\leftarrow Verify\left(PP,L,\mu ,\sigma \right)$: The vehicle uploads the signature and information to the RSU. The RSU verifies the validity of the signature, the verification process is as follows:

(1) For all of the $i\in \left[N\right]$, computes $t_{0}=-Az+\sum_{i\in \left[N\right]}pk_{i}\cdot g_{i}$ and $g=g_{1}⊗\ldots ⊗g_{N}$.
(2) If both $z\leftarrow S_{md^{2}-d}^{m}$ and $g=H\left(L,C,\mu ,t_{0}\right)$ are satisfied simultaneously, then the output is 1; otherwise, the output is 0.

$0/1\leftarrow Trace\left(PP,L,sk_{TA},\sigma \right)$: When the inquiry Institution finds that the message can be used as evidence, it initiates an identity verification application. Based on the tracking tag C in the signature, TA can compute with the private key $sk_{TA}=\left(s,e\right)$ to recover the identity tag $id_{s\pi }=\left[id_{\pi }|id_{s}\right]$ (see Section 2.2.3). According to $id_{s\pi }$, look up the corresponding $\left(pk_{\pi },sk_{\pi }\right)$ in the registration information form. If $pk_{\pi }\in L$, then outputs 1; otherwise, the outputs 0.

When the inquiry institution receives 1 sent back by TA and confirms the legitimacy of the identity, the message serves as evidence. Then, the signer can receive the corresponding reward through TA. If a false message is found, the responsibility can be pursued through TA. If the inquiry department receives 0 sent back by TA, the message will not be accepted.

## 4. Security Analysis

### 4.1. Correctness Proof

According to the signature algorithm, for an honestly generated signature $\sigma =\left(C,z,g_{1},\ldots g_{N}\right)$, we can get

(9) $$\begin{array}{l} t_{0}=-Az+\sum_{i\in \left[N\right]}pk_{i}\cdot g_{i}\\ =-Az+pk_{\pi }\cdot g_{\pi }+\sum_{i\in \left[N\right]\backslash \pi }pk_{i}\cdot g_{i}\\ =-A\cdot sk_{\pi }\cdot g_{\pi }+Ay+pk_{\pi }\cdot g_{\pi }+\sum_{i\in \left[N\right]\backslash \pi }pk_{i}\cdot g_{i}\\ =Ay+\sum_{i\in \left[N\right]\backslash \pi }pk_{i}\cdot g_{i} \end{array}$$

Therefore, when equations $z\leftarrow S_{md^{2}-d}^{m}$ and $g=H\left(L,C,\mu ,t_{0}\right)=g_{1}⊗\ldots ⊗g_{N}$ are being signed, they are already guaranteed to hold. Then, this condition must be satisfied when verifying the algorithm.

### 4.2. Security Proof

If the MSIS and S-MLWE problems are difficult to solve, then the algorithm proposed in this paper satisfies anonymity and unforgeability. For specific proof, please refer to Appendix A.

### 4.3. Traceability Proof

**Theorem** **1** (TA traceability)**.** *The identity tag can be correctly restored based on the tracking tag, and only TA can trace the signer’s identity.*

According to the MLWE-based PKC (see Section 2.2.3), only the TA’s private key can recover $id_{s\pi }$, and the correctness of this algorithm has been explained above. Since the small elements $\left(r,\varepsilon_{1},\varepsilon_{2}\right)$ in the tag are randomly generated each time, the value of tag C generated by each signature is different and cannot be linked.

Situation analysis: This signature method is different from others method that integrates identity into the private key. The private key and identity are completely independent and confidential. Even in the case of partial information leakage, it can still ensure that malicious nodes cannot generate valid signatures.

Suppose the identity tag obtained by TA is $i{d^{\prime }}_{s\pi }$. According to unforgeability, a signature cannot be carried out with only the public key. The signer must have a legitimate public–private key pair to perform the signature. If there is a situation where a malicious vehicle attempts to impersonate another person’s identity to sign, the specific analysis is as follows:

Case 1: If the signers’ keys $\left(pk_{\pi },sk_{\pi }\right)$ have not been leaked, then the malicious vehicle needs to sign with its own $\left(pk_{i},sk_{i}\right)$ but use someone else’s $i{d^{\prime }}_{s\pi }$ for the identity tag. Due to the inconsistency of the registration information, the verification of $Trace\left(PP,L,sk_{TA},\sigma \right)$ cannot be passed, and the output value is 0.

Case 2: When the information of the private key $sk_{\pi }$ is leaked, but the identity information $id_{\pi }$ is not. Because of $i{d^{\prime }}_{\pi }\neq id_{\pi }$, then $i{d^{\prime }}_{s\pi }\neq id_{s\pi }$, the signature cannot pass the verification of $Trace\left(PP,L,sk_{TA},\sigma \right)$, and the output value is 0.

Case 3: When the identity information $id_{\pi }$ is leaked but the information of the private key $sk_{\pi }$ is not, the malicious vehicle needs to sign with its own public–private keys $\left(pk_{i},sk_{i}\right)$. Because of $i{d^{\prime }}_{s}\neq id_{s}$, then $i{d^{\prime }}_{s\pi }\neq id_{s\pi }$, the signature cannot pass the verification of $Trace\left(PP,L,sk_{TA},\sigma \right)$, and the output value is 0.

Case 4: If both the identity information $id_{\pi }$ and the private key information $sk_{\pi }$ are leaked, and the malicious vehicle and vehicle π are in the same RSU range during signing, $Trace\left(PP,L,sk_{TA},\sigma \right)$ can pass. This situation needs two things: the leak of all signers’ information, the malicious vehicle, and the vehicle is in the same area during verification. So, the chance of this happening is negligible. If such a situation occurs, the only option is to wait for the inquiry institutions to discover it and then feed back the corresponding false information to TA. TA reviews the communication records of vehicle π to verify whether vehicle π has uploaded the information.

## 5. Performance Evaluations and Comparisons

In this chapter, we demonstrate the feasibility and superiority of the designed system through simulation and comparison. The experiments were implemented using MATLAB 2024a, running on a machine equipped with a 9th-generation Intel Core i7-9750H processor and 16 GB of RAM.

### 5.1. Parameter Selection and Size Comparison

Based on the algorithm in this paper, you can obtain the size for each part. The user’s public key is $pk_{i}\in R_{q}^{n}$, with $R_{q}={\mathbb{Z}}_{q}\left[x\right]/\left(x^{d}+1\right)$. The public key size is about ndlogq, using a base-2 logarithm.

Private key size: The user’s private key is $sk_{i}=\left(s_{i},e_{i}\right)\leftarrow S_{\beta }^{v}\times S_{\beta }^{n}$, and $S_{\beta }=\left\{f\in R_{q},\;{\left\|f\right\|}_{\infty }\leq \beta \right\}$, and the size is approximately $md\log\left(2\beta +1\right)$.

Signature size: According to the signature $\sigma =\left(C,z,g_{1},\ldots ,g_{N}\right)$, where $C=\left(C_{1},C_{2}\right)\in R_{q}^{v}\times R_{q}$, $z\leftarrow S_{md^{2}-d}^{m}$, $g_{i}\in D=\left\{g\in R_{q},\;{\left\|g\right\|}_{\infty }\leq 1\right\}$, the size of the signature is approximately $\left(v+1\right)d\log q+md\log\left(2md^{2}\right)+Nd\log3$.

When setting the design parameter values, the Root Hermite Factor δ needs to be considered. δ is a key parameter in lattice-based cryptography for evaluating the quality of a lattice basis or the difficulty of lattice problems. The smaller the value δ, the higher the quality of the lattice basis. Referring to the values δ≈1.0045 in [25], it can ensure 128-bit post-quantum security. Therefore, the value of parameters is considered. According to [22], the following two cases are mainly compared in Table 2.

128-bit security meets NIST (National Institute of Standards and Technology) level 1standard. It’s balanced quantum security and good performance, making it suitable for communication uses.

Although the lattice cryptography can resist quantum attacks, the large signature size and slow running time are the major issues, which impact its application effect, especially in ring signatures. The more the number of ring members, the stronger the concealment, but it will also lead to an increase in the signature size, occupying more storage space and computing consumption. Therefore, this paper mainly compares the changing trends of signature size and running time as the number of ring members increases.

By comparing the sizes under different parameters in Table 2, the first set of data is selected for simulation and comparison, as the growth rate of its signature size is relatively slow. And the sizes of public and private keys are not affected by the number of ring members; it has a relatively small storage overhead. This article selects the following schemes for comparison, under the condition of similar security bit lengths.

Wen et al. [21] designed a revocable ring signature for VANETs. It used lattice cryptography for identity verification and to tackle quantum security challenges. Ye et al. [22] proposed a traceable ring signature scheme for post-quantum blockchains, which significantly improved the size and runtime of the signature. Liu et al. [31] designed a linkable ring signature scheme that can stealthily address. Tang et al. [32] also designed an identity-based linkable ring signature scheme, and Hu et al. [33] proposed a linkable ring signature scheme with stronger security. References [32,33] differ from others in that they employ a lattice-based cryptography method based on trapdoors. Liang et al. [34] proposed a lattice-based certificateless traceable ring signature scheme. The characteristics of the above-mentioned scheme are shown in Table 3.

The results in Table 4 prove that using a DualRing structure can effectively reduce the size of the signature. The signature size generated by reference [22] and our proposals is significantly smaller than that of the other literature. References [21,31,34] still adopt a single-ring structure, and its size is much larger.

By comparison, it can be found that reference [21] and our proposals are most suitable for the IoV system. However, the number of ring members applicable in our proposals is 5–1000, while in reference [21] it is 5–8; thus, the application scope of our proposals is broader.

In terms of storage overhead, reference [22] is slightly better than ours, but the algorithm in reference [22] is linkable and not suitable for the IoV forensics system. Many ring signatures are publicly traceable. This means they meet both traceability and linkability, as shown in references [22,34]. Using the MLWE-based PKC ensures randomness and indistinguishability of tracking tag. So, it fits the Internet of Vehicles system better than the method in reference [22].

References [31,32,33,34] are not only not applicable to this system, but also the signature size is larger. References [32,33] indicate that trapdoor-based ring signature algorithms do not have an advantage in terms of signature size. As shown in Table 4, the signature size of ours is superior to most ring signature schemes.

### 5.2. Runtime

References [31,34] did not provide the running time. We compared the running times given in references [21,22,32]. The running times of the algorithms are shown in the following Table 5, Table 6 and Table 7. Reference [33] only provides the running time when the number of ring members is 2, which is much longer than other references, and thus has no comparative value. Reference [22] provided a complete MATLAB program. After running this program, we obtained the data in Table 6 and Table 7, and the algorithm running environment was consistent with ours. Since other references did not provide the algorithm programs, the running times were calculated based on the data they provided. 

**Figure 4:**
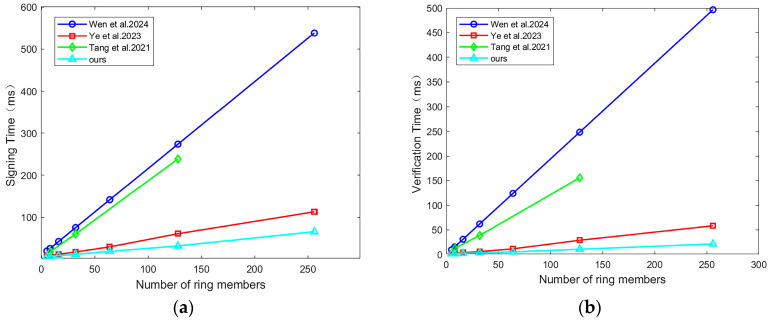
Comparison of the scheme proposed by [21,22,32], and ours in runtime: (**a**) Comparison of signing time; (**b**) Comparison of verification time.

In the IoV system, Setup, KeyGen, and TA Trace don’t happen during wireless communication between vehicles and RSUs. Therefore, they aren’t seen as key reference standards. According to the data comparison, this paper has made a significant improvement in the computation time. The maximum number of ring members is set to 8 in reference [21]; our runtime is only 28.6% and 11.4% of that in reference [21] under the same conditions. Reference [32] is a lattice-based cryptographic scheme with trapdoors, and the maximum number of ring members is given as 128. Under the same conditions, our runtime is only 13.4% and 7% of that in reference [32]. Due to the change in the simulation environment in reference [22], there are minor differences from the original text. When the number of ring members increases to 256, the runtime of this paper is only 58.2% and 36.8% of that in reference [22]. When the number of ring members increases to 1024, our signature time and verification time are 232 ms and 78 ms. Figure 4 more intuitively demonstrates the advantages of ours in terms of signature time and verification time.

According to Reference [35], in the IoV environment, when the number of vehicles is 40, the V2R communication delay does not exceed 3 ms. Combined with the algorithm in this paper, when the number of vehicles is 32, the process from vehicle signature to RSU verification will not exceed 19 ms. Collecting evidence is a time-consuming process, so the forensic system does not have high requirements for timeliness. The V2R communication delay has little impact on the system. So, this paper mainly compares algorithm performance.

## 6. Conclusions

This paper proposes an IoV forensics system based on post-quantum blockchain. Realizes anonymous forensics, distributed storage, and resistance to quantum attacks for IoV. The combination of the signature algorithm and the DualRing structure effectively reduces the size of the signature and accelerates computational speed. The MLWE-based PKC algorithm is used to encrypt the identity information, achieving the TA traceability and unlinkability of the tracking tag. Through the simulation of storage overhead and computing time, the efficiency of the improved algorithm and the application range of ring signatures are demonstrated. The results show that the structure in this paper meets IoV forensics needs. This system can also be applied to application environments with a large number of ring members, such as anonymous voting.

The algorithm in this article adheres to the relevant standards of PQC (Post-Quantum Cryptography) standardization released by NIST. The security and feasibility of the scheme are proved under the random oracle model. PQC is applicable to the field of communication encryption in the IoV.

Due to the relatively low security level of RSU nodes, this paper has some shortcomings. To balance anonymity and traceability, signature verification and identity verification are, respectively, completed by RSU nodes and TA. Meanwhile, considering the efficiency issue, TA verification is not conducted before the message is on the chain, but only after the message can be used as evidence. This leads to the possibility of false data on the chain, which is also the focus of the next improvement.

## Figures and Tables

**Table 1:** Relevant Parameters and Definitions.

Notation	Definition
ℤ	Integer set
${\mathbb{Z}}_{q}$	Integer set modulo odd prime q where the elements in ${\mathbb{Z}}_{q}$ are integers selected from the interval $\left[-\left(q-1\right)/2,\left(q-1\right)/2\right]$
R $,\;R_{q}$	The polynomial rings $R=\mathbb{Z}\left[x\right]/\left(x^{d}+1\right)$ $,\;R_{q}={\mathbb{Z}}_{q}\left[x\right]/\left(x^{d}+1\right)$
d	Degree of R $\;\mathrm{and}\;R_{q}$
A	Random matrix $A\leftarrow R_{q}^{n\times m}$ $,\;A=\left[A^{\prime }∥I_{n}\right]$
n,m,v	m and n are the column and row of A, v and n are the column and row of $A^{\prime }$
$I_{n}$	Identity matrix I with size n×n
H,D	$H:{\left\{0,1\right\}}^{*}\to D$ is a Hash function, output a set $D=\left\{g\in R_{q},\;{\left\|g\right\|}_{\infty }\leq 1\right\}$
$H^{\prime }$	$H^{\prime }:\left(\omega \right)\to {\left\{0,1\right\}}^{d/2}$ is a Hash function, output a binary string of length d/2
$S_{\beta }$	The set of $S_{\beta }=\left\{f\in R_{q},\;{\left\|f\right\|}_{\infty }\leq \beta \right\}$
N	Number of members in the ring $L=\left\{pk_{1},pk_{2},\ldots ,pk_{N}\right\}$
μ	The message needs to be signed
$sk_{TA},pk_{TA}$	The TA’s secret key $sk_{TA}=\left(s,e\right)$ , and TA’s public key $pk_{TA}=\left(A,b\right)$
$sk_{i},pk_{i}$	The secret key and public key of user $i\left(1\leq i\leq N\right)$
$id_{i}$	The user’s true identity of length d/2
ε	Error vector

**Table 2:** Parameter selection and dimensions.

Parameter Set	Set 1	Set 2
λ	128 bits	128 bits
q	≈$2^{26}$	≈$2^{26}$
d	128	256
m	15	7
n	7	3
β	1	1
pk	2.84 KB	2.44 KB
sk	0.37 KB	0.35 KB
σ	8.09 + 0.0247656N KB	8.39 + 0.049531N KB

**Table 3:** Comparison of requirements.

Requirements	Ours	21	22	31	32	33	34
anonymity	√	√	√	√	√	√	√
traceability	√	√	√	×	×	×	√
unlinkability	√	√	×	×	×	×	×
message integrity	√	√	√	√	√	√	√
Quantum resistance	√	√	√	√	√	√	√
λ	128	128	128	128	—	100	128

**Table 4:** Comparison of signature sizes (KB).

Number of Ring Members	N=5	N=8	N=64	N=128	N=256	N=1024
21	41.47	62.47	454.47	902.47	1798.47	7174.47
22	7.39	7.47	8.85	10.44	13.61	32.63
31	17.6	27.4	211.1	421.0	840.8	3359.9
32	36.98	60.91	471.8	943.46	1886.83	7547.04
33	—	5.1	38.7	78.1	157.3	625.8
34	4148	4654	6820	—	—	9709
Ours	8.21	8.29	9.67	11.26	14.43	33.45

**Table 5:** Operation Time of ours (ms).

Process	Setup	KeyGen	TA Trace
Ours	0.898	0.001	0.09

**Table 6:** Comparison of Signing Algorithm Runtime (ms).

Number of Ring Members	N=5	N=8	N=16	N=32	N=64	N=128	N=256
21	19.75	25.95	42.48	75.54	141.66	273.9	538.37
22	9.53	9.55	12.32	17.55	29.76	60.94	112.90
32	—	18.22	—	60.26	—	238.82	—
Ours	6.61	7.42	9.09	12.14	19.00	31.90	65.76

**Table 7:** Comparison of Verification Algorithm Runtime (ms).

Number of Ring Members	N=5	N=8	N=16	N=32	N=64	N=128	N=256
21	9.69	15.51	31.02	62.04	124.09	248.18	496.36
22	2.69	2.97	3.81	6.07	11.64	29.03	58.35
32	—	12.48	—	38.72	—	155.62	—
Ours	1.56	1.77	2.48	3.52	5.44	10.79	21.46

## Data Availability

Data are contained within the article.

## Abbreviations

The following abbreviations are used in this manuscript:

IoV	Internet of Vehicles
VANETs	Vehicular Ad Hoc Networks
V2V	Vehicle-to-Vehicle
V2I	Vehicle-to-Infrastructure
V2R	Vehicle-to-RSU
TA	Trusted Authority
RSUs	Road Side Units
RSU	Road Side Unit
OBUs	On-Board Units
RLWE	Ring Learning with Errors
MLWE	Module Learning with Errors
D-MLWE	Decisional MLWE
S-MLWE	Search MLWE
MSIS	Module Short Integer Solution
PKC	Public Key Cryptosystem
IND-CPA	Indistinguishability under Chosen Plaintext Attack
Type-T	Three-Move type
TRS	Traceable Ring signature
PQC	Post-Quantum Cryptography
NIST	National Institute of Standards and Technology

## Appendix A

### Appendix A.1. Security Proof

#### Appendix A.1.1. Signature Simulation

Under the random oracle model, combined with the algorithm designed in this paper, the challenger S creates three lists $L_{H}$, $L_{K}$, and $L_{S}$ to store the information that has already been queried. The specific query process is as follows:

Random Oracle H: S randomly selects a vector g from the set $D=\left\{g\in R_{q},\;{\left\|g\right\|}_{\infty }\leq 1\right\}$ and looks it up in the list $L_{H}$, which is used for storing g that has been asked before. If the g already exists in $L_{H}$, a random selection is performed again. If the g is not in the list, output g and save it to $L_{H}$.

Key Generation Oracle $O_{K}$: The adversary A can submit $id_{i}$ to S, S checks the list $L_{K}$, which is used for storage $\left(pk_{i},sk_{i},id_{si}\right)$. If the corresponding information $\left(pk_{i},sk_{i},id_{si}\right)$ already exists in the list, it will be returned to A. If it does not exist in the list $L_{K}$, S randomly generates $\left(pk_{i},sk_{i},id_{si}\right)$ according to the generation algorithm and saves it in the list $L_{K}$, and the information will be returned to A.

Signing Oracle $O_{S}$: A input the ring $L=\left\{pk_{1},pk_{2},\ldots ,pk_{N}\right\}$ and the signer’s public key $pk_{\pi }\in L$, the message μ, and S checks the list $L_{S}$, which is used for storage $\left(pk_{i},L,\mu ,\sigma \right)$. If it is in the list, S returns σ to A. Otherwise, S checks the corresponding information $\left(pk_{\pi },sk_{\pi },id_{s\pi }\right)$ through $pk_{\pi }$, S runs the algorithm to generate the signature σ, saves it in the list $L_{S}$, then returns σ to A.

When the signer possesses the corresponding $sk_{\pi }$ and $id_{s\pi }$, the ring signature can be carried out normally according to the content of Section 3.3.

The signer without the corresponding secret key and identity needs to reprogram H to obtain g to ensure that the verification algorithm passes. The signature process is as follows:

$\sigma \leftarrow Sign\left(PP,L,\mu \right)$: The signer uses $A^{\prime }$ provided by S to sign with a fake identity $id_{s\pi }^{*}$.

(1) Randomly selects the following small elements $\left(r,\varepsilon_{1},\varepsilon_{2}\right)\leftarrow S_{\beta }^{n}\times S_{\beta }^{v}\times S_{\beta }$, and computes $C=\left(C_{1},C_{2}\right)=\left({A^{\prime }}^{T}r+\varepsilon_{1},b^{T}r+\varepsilon_{2}+q/2\cdot id_{s\pi }^{*}\right)$ as a tracking tag.
(2) Samples $y\leftarrow S_{md^{2}}^{m},g_{i}\leftarrow D$ and $i\in \left[N\right]$.
(3) Computes $t_{0}=-Az+\sum_{i\in \left[N\right]}pk_{i}\cdot g_{i}$ and $g=g_{1}⊗\ldots ⊗g_{N}$.

If g has been answered by Random Oracle H before, return to (2) and start over.

Otherwise, reprogram H to obtain the output of $g=H\left(L,C,\mu ,t_{0}\right)$. Then, output the signature $\sigma =\left(C,z,g_{1},\ldots ,g_{N}\right)$.

#### Appendix A.1.2. Anonymity Proof

**Theorem** **A1** (Anonymity)**.** *If the problem of S-MLWE is hard, then the ring signature scheme proposed in this paper is anonymous in the random oracle model.*

**Proof.** According to the game rules between A and S, let $Game_{0}$ represent the signature process when B=0, let $Game_{1}$ represent the signature process when B=1. S sets the system parameters. According to the definition of anonymity, it can be known that each time A queries $O_{K}$, A will receive an instance $\left(pk_{i},sk_{i},id_{si}\right)$. After enough inquiries, a collection $L=\left\{pk_{1},pk_{2},\ldots ,pk_{N}\right\}$ can be generated. A inputs two valid public keys, $pk_{i0}$ and $pk_{i1}$, S randomly selects $B\in \left\{0,1\right\}$ and generates a signature according to the signature algorithm, and returns it to A. A makes a judgment on the value of B after receiving the signature. The anonymity of this scheme is proved by demonstrating the indistinguishability of the signatures $\sigma^{0}=\left(C^{0},z^{0},g_{1}^{0},\ldots ,g_{N}^{0}\right)$ generated by $Game_{0}$ and $\sigma^{1}=\left(C^{1},z^{1},g_{1}^{1},\ldots ,g_{N}^{1}\right)$ generated by $Game_{1}$.  □

$g_{i}^{0}\leftarrow D$ and $g_{i}^{1}\leftarrow D$, $z^{0}$ and $z^{1}$ are all randomly selected from the same space. Because the equation $z=sk_{\pi }g_{\pi }-y$ satisfies the rejection sampling theorem [23], the statistical distance between $z^{0}$ and $z^{1}$ is indistinguishable.

S signs with its own public key b; it is impossible to calculate the tracking tag without the private key s. The difficulty is equivalent to the problem of S-MLWE. For tracking tag $C=\left(C_{1},C_{2}\right)=\left({A^{\prime }}^{T}r+\varepsilon_{1},b^{T}r+\varepsilon_{2}+q/2\cdot id_{s\pi }\right)$ , we need to distinguish the differences between $C_{1}^{0}={A^{\prime }}^{T}r^{0}+\varepsilon_{1}^{0}$ and $C_{1}^{1}={A^{\prime }}^{T}r^{1}+\varepsilon_{1}^{1}$, and the differences between $C_{2}^{0}=b^{T}r^{0}+\varepsilon_{2}^{0}+q/2\cdot id_{si0}$ and $C_{2}^{1}=b^{T}r^{1}+\varepsilon_{2}^{1}+q/2\cdot id_{si1}$ . Compute $C_{2}^{0}-C_{2}^{1}=b^{T}\left(r^{0}-r^{1}\right)+\varepsilon_{2}^{0}-\varepsilon_{2}^{1}+q/2\left(id_{si0}-id_{si1}\right)$ , because r and ε are taken randomly. Then, $\left(r^{0}-r^{1}\right)$ and $\left(\varepsilon_{2}^{0}-\varepsilon_{2}^{1}\right)$ are still independently distributed, and $\left(id_{si0}-id_{si1}\right)$ has no correlation, so $C_{2}^{0}$ and $C_{2}^{1}$ cannot be distinguished. Similarly, $C_{1}^{0}$ and $C_{1}^{1}$ cannot be distinguished, so the signatures $\sigma^{0}$ and $\sigma^{1}$ are indistinguishable, and it can be proved that the scheme satisfies complete anonymity.

#### Appendix A.1.3. Unforgeability Proof

**Theorem** **A2** (Unforgeability)**.** *If the problems MSIS and S-MLWE are difficult, then the algorithm proposed in this paper is unforgeable in the random oracle model.*

**Proof.** According to the requirement of unforgeability, suppose A can forge a valid signature $\sigma^{\prime }=\left(C,z^{\prime },{g^{\prime }}_{1},\ldots ,{g^{\prime }}_{N}\right)$ for the message μ with a non-negligible probability ∂, while satisfying the following three conditions: S verifies that the signature is valid; A has not inquired about the private key of any member in the ring; A has not inquired about the signature of $\left(L,\mu \right)$.  □

To satisfy the above conditions, A needs to forge a signature according to the signature algorithm without the private key and identity information. Suppose that A can perform a maximum of $q_{H}$ random Oracle H queries. According to the General Forking Lemma [36], A can generate two signatures $\sigma^{*}=\left(C,z^{*},g_{1}^{*},\ldots ,g_{N}^{*}\right)$ and $\sigma^{\prime }=\left(C,z^{\prime },{g^{\prime }}_{1},\ldots ,{g^{\prime }}_{N}\right)$ for the same message μ with a probability at least $\partial^{2}/q_{H}$. Since the identity of the signer remains unchanged, the tracking tag can be set to remain unchanged. For the same input to $\left(L,C,\mu ,t_{0}\right)$, the Random Oracle H query, the output is $g^{\prime }\neq g^{*}$. So there is

(A1) $$t_{0}=-A\cdot z^{\prime }+\sum_{i\in \left[N\right]}pk_{i}\cdot {g^{\prime }}_{i}=-A\cdot z^{*}+\sum_{i\in \left[N\right]}pk_{i}\cdot g_{i}^{*}$$

Since

(A2) $$\begin{array}{l} -A\cdot z^{\prime }+\sum_{i\in \left[N\right]}pk_{i}\cdot {g^{\prime }}_{i}\\ =-A\cdot \left(z^{\prime }-sk_{1}\cdot {g^{\prime }}_{1}-\ldots -sk_{\pi -1}\cdot {g^{\prime }}_{\pi -1}-sk_{\pi +1}\cdot {g^{\prime }}_{\pi +1}-\ldots -sk_{N}\cdot {g^{\prime }}_{N}\right)+pk_{\pi }\cdot {g^{\prime }}_{\pi }\\ =-A\cdot \tilde{z^{\prime }}+A\cdot sk_{\pi }\cdot {g^{\prime }}_{\pi } \end{array}$$

and

(A3) $$\begin{array}{l} -A\cdot z^{*}+\sum_{i\in \left[N\right]}pk_{i}\cdot g_{i}^{*}\\ =-A\cdot \left(z^{*}-sk_{1}\cdot g_{1}^{*}-\ldots -sk_{\pi -1}\cdot g_{\pi -1}^{*}-sk_{\pi +1}\cdot g_{\pi +1}^{*}-\ldots -sk_{N}\cdot g_{N}^{*}\right)+pk_{\pi }\cdot g_{\pi }^{*}\\ =-A\cdot {\tilde{z}}^{*}+A\cdot sk_{\pi }\cdot g_{\pi }^{*} \end{array}$$

For the two equations to be equal, it is necessary that

(A4) $$A\cdot \left(sk_{\pi }\cdot g_{\pi }^{*}-{\tilde{z}}^{*}\right)=A\cdot \left(sk_{\pi }\cdot {g^{\prime }}_{\pi }-\tilde{z^{\prime }}\right)$$

(1) When $sk_{\pi }\cdot g_{\pi }^{*}-{\tilde{z}}^{*}=sk_{\pi }\cdot {g^{\prime }}_{\pi }-\tilde{z^{\prime }}$, equal to $sk_{\pi }\cdot \left({g^{\prime }}_{\pi }-g_{\pi }^{*}\right)=\tilde{z^{\prime }}-{\tilde{z}}^{*}$. Since $g^{\prime }\neq g^{*}$, ${g^{\prime }}_{i}$, and $g_{i}^{*}$ cannot all be equal, the probability that ${g^{\prime }}_{\pi }\neq g_{\pi }^{*}$ is at least 1/N. It can be obtained that $sk_{\pi }=\left(\tilde{z^{\prime }}-{\tilde{z}}^{*}\right){\left({g^{\prime }}_{\pi }-g_{\pi }^{*}\right)}^{-1}$ is equivalent to finding a solution to the problem of S-MLWE.
  That is to say, S can solve the problem S-MLWE with a probability of at least ∂2N⋅qH. This is contradictory, then $Adv_{A}^{forge}$ is negligible, and the algorithm satisfies unforgeability.
(2) When $sk_{\pi }\cdot g_{\pi }^{*}-{\tilde{z}}^{*}\neq sk_{\pi }\cdot {g^{\prime }}_{\pi }-\tilde{z^{\prime }}$, we can obtain a non-zero vector solution $e=\left(sk_{\pi }\cdot g_{\pi }^{*}-{\tilde{z}}^{*}\right)-\left(sk_{\pi }\cdot {g^{\prime }}_{\pi }-\tilde{z^{\prime }}\right)$ that satisfies the condition Ae=0, which is equivalent to finding a solution to the MSIS problem.

That is to say, S can solve the problem MSIS with a probability of at least ∂2qH. This is contradictory, then $Adv_{A}^{forge}$ is negligible, and the algorithm satisfies unforgeability.
